# Challenges of monocyte HLA-DR targeted immunomodulation in sepsis—a prospective observational cohort study

**DOI:** 10.3389/fimmu.2025.1709289

**Published:** 2026-01-07

**Authors:** Timothy Arthur Chandos Snow, Antoine Villa, Antonio Cesar, Francis Ryckaert, Naveed Saleem, Deborah Smyth, Holly Pan, Julia Flint, David Brealey, Mervyn Singer, Derek W. Gilroy, Nishkantha Arulkumaran, the University College London Hospitals Critical Care Research Team

**Affiliations:** 1 Bloomsbury Institute of Intensive Care Medicine, Division of Medicine, University College London, London, United Kingdom; 2 Department of Ageing, Rheumatology and Regenerative Medicine, Division of Medicine, University College London, London, United Kingdom; 3 UCLH NIHR Biomedical Research Centre, University College London Hospitals NHS Foundation Trust, London, United Kingdom

**Keywords:** antigens, HLA-DR antigens, immunotherapy, infections, interferon-gamma, monocytes, sepsis

## Abstract

**Introduction:**

Reduced monocyte HLA-DR expression, a hallmark of immunosuppression in sepsis, is associated with infectious complications and mortality. Therapeutic strategies, including IFN-γ, have been used to restore monocyte HLA-DR and immune function, but have not consistently improved clinical outcomes. Therefore, we conducted an iterative series of experiments to re-examine the core assumptions and address the key gaps in the current understanding.

**Methods:**

We conducted a prospective cohort study of patients admitted to the intensive care unit (ICU) with sepsis (n = 55, 36% mortality) to characterize the dynamics of monocyte HLA-DR expression and associated functional pathways. Flow cytometry was used to evaluate monocyte phenotype, and lipopolysaccharide (LPS) stimulation was used to assess monocyte functional capacity. We examined canonical monocyte pathways and identified those that were responsive to LPS stimulation and/or modulated by IFN-γ *ex vivo*. We evaluated monocyte HLA-DR expression in the peripheral blood and inflamed tissues of healthy volunteers following intradermal administration of UV-killed *E. coli*.

**Results:**

Monocyte HLA-DR expression was significantly lower in patients than in healthy volunteers, particularly in non-survivors. Monocyte phenotypes evolved discordantly over time, some markers trended toward healthy levels, while others diverged, with no consistent distinction between survivors and non-survivors. Intracellular trafficking of membrane HLA-DR on bacterial phagocytosis contributes to the reduced surface HLA-DR expression. Compared to healthy volunteers, monocytes from ICU patients had a significantly lower expression of proteins associated with antigen presentation and co-stimulation, cytokines, phagocytosis, and a blunted response to LPS. IFN-γ increased the levels of proteins involved in antigen presentation, but their expression remained significantly lower than that in healthy controls. Healthy volunteers demonstrated compartment-specific and temporally distinct regulation of monocyte HLA-DR in circulation versus that in inflamed tissue.

**Conclusion:**

Reduced monocyte HLA-DR expression in sepsis reflects broad disruptions across multiple pathways, explaining the limited efficacy of therapeutic interventions. Further insights into the mechanisms governing therapeutic modulation of monocyte HLA-DR and immune function are required to identify patients who are most likely to benefit from intervention.

## Introduction

Sepsis, a life-threatening organ dysfunction due to a dysregulated host response to infection (1), is responsible for 50 million deaths per annum globally (accounting for 20% of all deaths) (2). A key strategy for managing bacterial infections involves host-directed therapies aimed at enhancing protective immune responses. The need for such therapies in sepsis is critical, as many patients die from persistent and/or secondary infections associated with sepsis-induced immunosuppression (3, 4).

Reduced monocyte HLA-DR expression is a well-established marker of immunosuppression and is associated with an increased risk of secondary infections and mortality in patients presenting to the Emergency Department (ED) with infections (5), critically ill patients with sepsis (3, 6–9), and those following major trauma or surgery (10–12). Therapeutic interventions that increase monocyte HLA-DR expression include granulocyte colony-stimulating factor (G-CSF), granulocyte–macrophage colony-stimulating factor (GM-CSF), and interferon-gamma (IFN-γ). Pilot studies have demonstrated the potential benefits of G-CSF/GM-CSF treatment in critically ill patients (13–18). However, in a recent clinical trial, GM-CSF had no effect on the prevention of ICU-acquired infection in sepsis, but any conclusion was limited by the early termination of the study, leading to a low number of included patients (19). Similarly, recombinant human IFN-γ has been associated with improved surrogates of immune function in preliminary studies (20–22). However, a recent randomized control trial of IFN-γ for the prevention of hospital-acquired pneumonia in critically ill patients was terminated early because of the potential harm caused by IFN-γ treatment (23).

To address the discrepancy between promising early clinical studies underpinned by biological plausibility yet lack of benefit in larger clinical trials, we conducted a series of experiments to revisit foundational assumptions and address key gaps in the current understanding.

First, we assessed the longitudinal evolution of monocyte HLA-DR in relation to associated immune markers over a 5-day period to determine whether immunosuppression markers evolve in tandem and whether these trajectories differ between survivors and non-survivors.

A limitation of prior studies is their reliance on static immune profiling, such as RNA (transcriptomic or PCR) or protein-level (flow cytometry) analyses, which may not reflect functional immune competence. We propose that dynamic changes in immune cell phenotype in response to stimuli (e.g., lipopolysaccharide (LPS) or live bacteria) or therapeutic agents (e.g., IFN-γ) provide a more accurate representation of immune function.

Therefore, we assessed the functional capacity of monocytes in response to LPS and IFN-γ. Specifically, we measured canonical monocyte cytokine responses (IL-1β, TNF-α, and IL-10) following *ex vivo* LPS stimulation and examined their associations with illness severity and intracellular cytokine levels. Next, we examined the expression of multiple canonical monocyte functional pathways in sepsis, identifying those that responded to LPS stimulation and/or were modulated by IFN-γ *ex vivo* as a therapeutic agent.

Although reduced monocyte HLA-DR expression is widely considered a hallmark of immunosuppression in critical illness, it also occurs, albeit to a lesser extent, in patients presenting early to the emergency department with milder infections. We evaluated monocyte HLA-DR trafficking immediately before and after bacterial phagocytosis *ex vivo*, by comparing healthy volunteers and ICU patient monocytes.

Another limitation in our understanding of monocyte biology is the near-exclusive focus on peripheral blood, with limited insight into how well this reflects the monocyte phenotype at sites of infection or inflammation (‘compartmentalization’ of the immune response in sepsis). To address this, we assessed temporal monocyte HLA-DR expression in both peripheral blood and inflamed tissue of healthy volunteers following intradermal administration of UV-killed E. *coli*.

## Materials and methods

### Study design, ethical approval, and participants

We conducted a prospective observational cohort study recruiting patients aged ≥18 years who presented to the ICU at University College London Hospitals (UCLH) between 1 August 2021 and 26 January 2023, and had blood cultures taken for suspicion of bacterial infection. The exclusion criteria for this study included patients with severe anemia and contraindication to blood transfusion, those not expected to survive beyond 24 h of admission, and pregnant women.

Patient demographics, clinical data (physiology and diagnoses), laboratory data, and clinical outcomes were recorded from electronic healthcare records. Patients were followed up until hospital discharge or death. This study was conducted in parallel with a study investigating biomarkers of sepsis (REC reference 20/LO/1024). Venous blood samples were collected from a group of healthy volunteers between August 2021 and January 2023. Approval was obtained from the University College London (UCL) REC, reference 19181/001.

Based on our previous data in surgical patients (24) demonstrating a monocyte HLA-DR median fluorescence intensity (MFI) of 5,000 ± 1,250, with a power of 80% and alpha of 0.05, a sample size of ≥25 patients per group was included to detect a statistically significant difference of 20% between groups.

In a separate study, monocyte HLA-DR expression was simultaneously characterized in blood and inflammatory tissue at different time points using an intradermal UV-killed E. *coli* blister model in healthy volunteers (25) (UCL REC reference 1309/005).

### Sample processing

Whole blood was collected in CPT™ (8 ml), K2 EDTA (4 ml), and SST Advance™ vacutainers (4 ml, all Becton Dickinson (BD), Wokingham, UK) and processed within 30 min. CPT vacutainers were centrifuged at 1,500*g* for 15 min, and the peripheral blood mononuclear cell (PBMC) layer was extracted, washed twice in phosphate-buffered saline (PBS), resuspended in freezing media (fetal bovine serum (FBS) (Gibco, Thermo Fisher, Cambridge, UK) with 10% dimethyl sulfoxide (DMSO) (Sigma-Aldrich (Sigma), Gillingham, UK)), and cooled to −80°C using an isopropyl alcohol gradient (Mr Frosty™, TF). After 24 h–48 h, the PBMCs were transferred for storage in liquid nitrogen. Frozen PBMCs were rapidly defrosted in batches using media, washed twice in media, and either stained or rested for 1 h prior to stimulation. Serum was stored at −80°C.

### *Ex vivo* whole blood stimulation

Whole blood cytokine release was assessed within 30 min of collection by mixing 500 μl EDTA whole blood with 100 ng/mL LPS (Sigma) and incubating for 1 h at 37°C and 5% CO_2_. The samples were then centrifuged at 1,500*g* for 5 min, and the serum was stored at −80°C for subsequent analysis.

### *Ex vivo* PBMC stimulation

We investigated the ability of monocytes isolated from critically ill patients to respond to pHrodo (100 mcg/mL) (Thermo Fisher) opsonized bioparticles for 1 h at 37°C and 5% CO_2_.

Separately, we investigated the ability of monocytes from critically ill patients and healthy volunteers to respond to 100 ng/mL LPS (Sigma-Aldrich) and/or 100 ng/mL IFN-γ (Sigma-Aldrich) for 24 h at 37°C and 5% CO_2_. The doses were identified through the existing literature (26) and preliminary experiments (**Supplementary Figure S1**).

### UV-killed *E. coli* blister model

The local and systemic immune cell phenotypes were investigated as part of an independent study. However, this provided an opportunity to evaluate the concurrent expression of peripheral blood and inflammatory tissue monocyte HLA-DR. UV-killed *E. coli* (5 ng) was diluted in sterile saline (50 μl) and intradermally injected into two marked sites on the ventral side of the right forearm of eight female participants. After 4 h and 24 h, a suction blister was used to remove the inflammatory exudate from one of the LPS-exposed injection sites.

Blister fluid was collected in a V-bottom plate containing 50 μl of 3% sodium citrate (Sigma) in PBS and kept on ice. Peripheral PBMCs were isolated at the same time points to allow for direct comparison. PBMCs and blister cells were centrifuged, resuspended in RoboSep buffer (Stemcell, Vancouver, Canada), and stained with cell surface markers.

### Flow cytometry

Conventional flow cytometry was used for baseline immunophenotyping of the critically ill cohort. PBMCs were centrifuged and resuspended in cell staining buffer (BioLegend, London, UK) containing fluorochrome-labeled antibodies against the following markers (CD14, CD16, HLA-DR, CD80, CD86, and CD274 (PD-L1)) and viability stain (Live/Dead). After using the Cytofix/perm fixation/permeabilization kit (BD) as per the manufacturer’s recommendations, fluorochrome-labeled antibodies against the following intracellular cytokines (IL-1β, IL-6, IL-10, and TNF-α) were used. The full details of the antibodies and their concentrations used are provided in **Supplementary Table S1**. Concentrations were determined using dose titrations as recommended by the manufacturer. Cells were acquired on an LSRII flow cytometer (BD) running FACSDiva™ software (version 9, BD), and calibration beads (BD) were used prior to running each experiment.

Detailed immunophenotyping was performed using spectral flow cytometry in a subset of healthy volunteers and ICU patients. PBMCs were centrifuged and resuspended in cell staining buffer with fluorochrome-labeled antibodies against the following markers (CD14, CD16, HLA-DR, CD64, CD74, CD80, CD86, CD192, CD274, CD284, HLA-DM, and HLA-DP) and viability stain (Live/Dead). After using the Tru-nuclear fixation/permeabilization kit (BioLegend) as per manufacturer’s recommendations, fluorochrome-labeled antibodies against the following intracellular cytokines (IL-1β, IL-10, IFN-γ, and TNF-α), proteins (NF-κB, NLRP3, NOX-2), and transcription factors (CIITA) were used. The full details of the products and concentrations used are listed in **Supplementary Table S2**. Cells were acquired using an ID7000 spectral cell analyzer (Sony Biotechnology Inc., Weybridge, UK) running the ID7000 software (version 1.2, Sony). Alignment check beads (Sony) were used prior to each experiment.

Conventional flow cytometry was used for the *in vivo* UV-killed *E. coli* blister model. Cells were stained with fluorochrome-labeled antibodies for the following markers (CD14, CD16, and HLA-DR) and analyzed using a MACSQuant 10 (Miltenyi Biotec GmbH (MB), Bergisch Gladbach, Germany) flow cytometer and Flowlogic 7.1 (Inivai, Mentone, Australia). The product details are provided in **Supplementary Table S3**.

Compensation controls or spectral references for each fluorochrome were added using either single-stain labeled cells (pHRodo), heat-killed cells (60°C for 10 min, viability stains), or compensation beads (BD or Thermo Fisher) with appropriate negative controls. Fluorescence minus one (FMO) samples were used to identify cell populations. The stopping gate was set at 10,000 events for HLA-DR^+^ CD14^+^CD16^-^ monocytes. An example gating strategy for both conventional and spectral flow cytometry is shown in **Supplementary Figure S2**.

### Serum and supernatant cytokine measurements

C-reactive protein (CRP) levels were measured in the hospital laboratory. IL-1β, IL-10, and TNF-α levels were measured in serum or cell culture supernatant using Duoset ELISA kits (R&D Systems, Minneapolis, MN, USA) according to the manufacturer’s instructions. The samples were diluted 1:10 in a reagent diluent. Optical densities were measured using a SPECTROstar Nano microplate reader (BMG Labtech, Aylesbury, UK).

### Confocal microscopy

Immune cells were imaged using an inverted confocal microscope (Zeiss LSM 880) with a ×63 1.4 oil immersion objective. Immune cells were plated on 1 mm slides (Academy microscope slides). Imaging was performed within 24 h of isolation. During imaging, the cells were maintained in phosphate-buffered saline (or serum, if stated). To identify monocytes, cells were incubated for 30 min at 37°C with an APC-conjugated anti-CD14 antibody (BioLegend), BV711-conjugated anti-HLA-DR antibody (BioLegend), LysoTracker™ Green DND-26 (Invitrogen) for lysosomal identification, and Hoechst 33342 (Invitrogen) for nuclear visualization. To confirm the internalization of HLA-DR fluorescence during phagocytosis, cells were loaded for 30 min at 37°C with 0.1 mg/ml pHrodo™ Red *E. coli* BioParticles™ (Invitrogen). Imaging was performed immediately after loading, with excitations set at 405 nm for Hoechst 33342, 488 nm for LysoTracker™ Green, 561 nm for pHrodo ™ Red *E. coli* BioParticles, 633 nm for HLA-DR-BV711, and 594 nm for CD14-APC. The detection wavelengths were 415 nm–496 nm for Hoechst 33342, 495 nm–584 nm for LysoTracker™ Green, 566 nm–584 nm for pHrodo ™ Red *E. coli* BioParticles, 694 nm–759 nm for HLA-DR-BV711, 649 nm–691 nm for CD14-APC. Image processing was performed using Zeiss Zen lite software. The proportion of internalized HLA-DR to whole-cell HLA-DR expression was quantified by measuring the fluorescence intensity of the whole cell or internal two-thirds of the cell using the Fiji software.

### Statistics

Statistical analyses were performed and graphs were constructed using Prism (Version 10, GraphPad, San Diego, CA, USA). Continuous and categorical variables are reported as median (interquartile range) and n (%), respectively.

Categorical data were compared using the chi-square test. Continuous data were compared using Wilcoxon or Mann–Whitney tests for paired and unpaired data, respectively. Differences between three or more groups were compared using Friedman or Kruskal–Wallis tests with Dunn’s uncorrected tests for paired and unpaired data, respectively.

To ascertain differences between healthy volunteers and ICU patient monocyte response following *ex vivo* LPS and/or IFN-γ stimulation, a principal component analysis (PCA) was performed, as were multiple comparisons performed using a Mann–Whitney test and volcano plots generated using a corrected p-value (−log10) with a False Discovery Rate (FDR) of 5% calculated using the two-stage step-up method of Benjamini, Krieger, and Yekutieli.

## Results

### Clinical data

A total of 55 patients were included, with a hospital mortality rate of 36% (**Table 1**, **Supplementary Figure S3**). The median age was 55 (42–65) years, and 55% were male. There were no differences in co-morbid illness between survivors and non-survivors. A greater proportion of non-survivors had respiratory infections (p = 0.044). The overall illness severity score (SOFA score) on admission was significantly higher among non-survivors (p = 0.0003); however, there were no differences in the use of mechanical ventilation, cardiovascular support (vasopressors or inotropes), or renal replacement therapy between the two groups. No differences in immune cell counts were observed between survivors and non-survivors. Healthy volunteers (n = 16) included 11 (67%) males, aged 36 (35–38) years.

**Table 1:** Demographics, clinical, and laboratory data of patients included in the study.

Variable	ICU patients (n = 55)	Survivor (n = 35)	Non-survivor (n = 20)	p-value
Age	55 (42–65)	55 (40–64)	55 (47–70)	0.3427
Male (%)	32 (58%)	19 (35%)	13 (65%)	0.150
Co-morbid illness				
Diabetes mellitus	10 (18%)	7 (20%)	3 (15%)	0.731
Ischaemic heart disease	5 (9%)	1 (3%)	4 (20%)	0.053
COPD	5 (9%)	2 (6%)	3 (15%)	0.324
Active Cancer	20 (36%)	11 (31%)	9 (45%)	0.387
Hemato-oncology	16 (29%)	8 (23%)	8 (40%)	0.224
Infection source				
Lung	22 (40%)	10 (29%)	12 (60%)	0.044
Abdo	16 (29%)	11 (31%)	5 (25%)	0.761
Urine	1 (2%)	1 (3%)	0 (0%)	0.999
Other (Line, sinus, soft tissue, CNS)	13 (24%)	10 (29%)	3 (15%)	0.333
Not identified	3 (5%)	3 (5%)	0 (0%)	0.293
Organ support				
Mechanical ventilation HFNO NIV/CPAP	29 (52%) 9 (16%) 2 (4%)	16 (46%) 6 (17%) 0 (0%)	13 (65%) 3 (15%) 2 (10%)	0.794 0.999 0.128
Cardiovascular	14 (25%)	6 (17%)	8 (40%)	0.106
Renal replacement therapy	7 (13%)	5 (14%)	2 (10%)	0.999
SOFA score	6 (4–8.5)	5 (4–7)	8 (6–13)	0.0003
Laboratory tests				
CRP (mg/l)	185 (80–252)	199 (129–242)	138 (21–282)	0.462
Bilirubin (μmol/l)	13 (7–27)	13 (5–21)	16 (8–49)	0.171
Creatinine (μmol/l)	79 (48–132)	75 (50–108)	97 (42–142)	0.655
Lactate (mmol/l)	1.0 (0.8–2.0)	0.8 (0.7–1.5)	1.4 (0.9–3.2)	0.044
Platelet (×10^9^)	171 (54–261)	207 (107–270)	78 (35–172)	0.010
WBC (×10^6^)	9.3 (5.1–12.7)	10.2 (6.1–12.7)	7.7 (1.4–12.9)	0.180
Lymphocyte (×10^6^)	0.84 (0.40–1.43)	0.93 (0.56–1.62)	0.69 (0.12–1.43)	0.184
Neutrophil (×10^6^)	7.3 (4.1–9.8)	7.8 (4.8–9.7)	5.7 (0.9–11.4)	0.205
NLR	7.6 (4.5–14.2)	8.4 (4.4–13.8)	6.4 (4.5–14.8)	0.803
Monocyte (×10^6^)	0.5 (0.2–0.9)	0.7 (0.2–1.0)	0.4 (0.1–0.9)	0.165

COPD, chronic obstructive pulmonary disease; CNS, central nervous system; CRP, C-reactive protein; ICU, intensive care unit; SOFA, Sequential Organ Failure Assessment; NLR, neutrophil:lymphocyte ratio; WBC, white blood cell. P-value signifies difference between survivors and non-survivors.

### Monocyte phenotype in sepsis survivors and non-survivors

**Figure 1:**
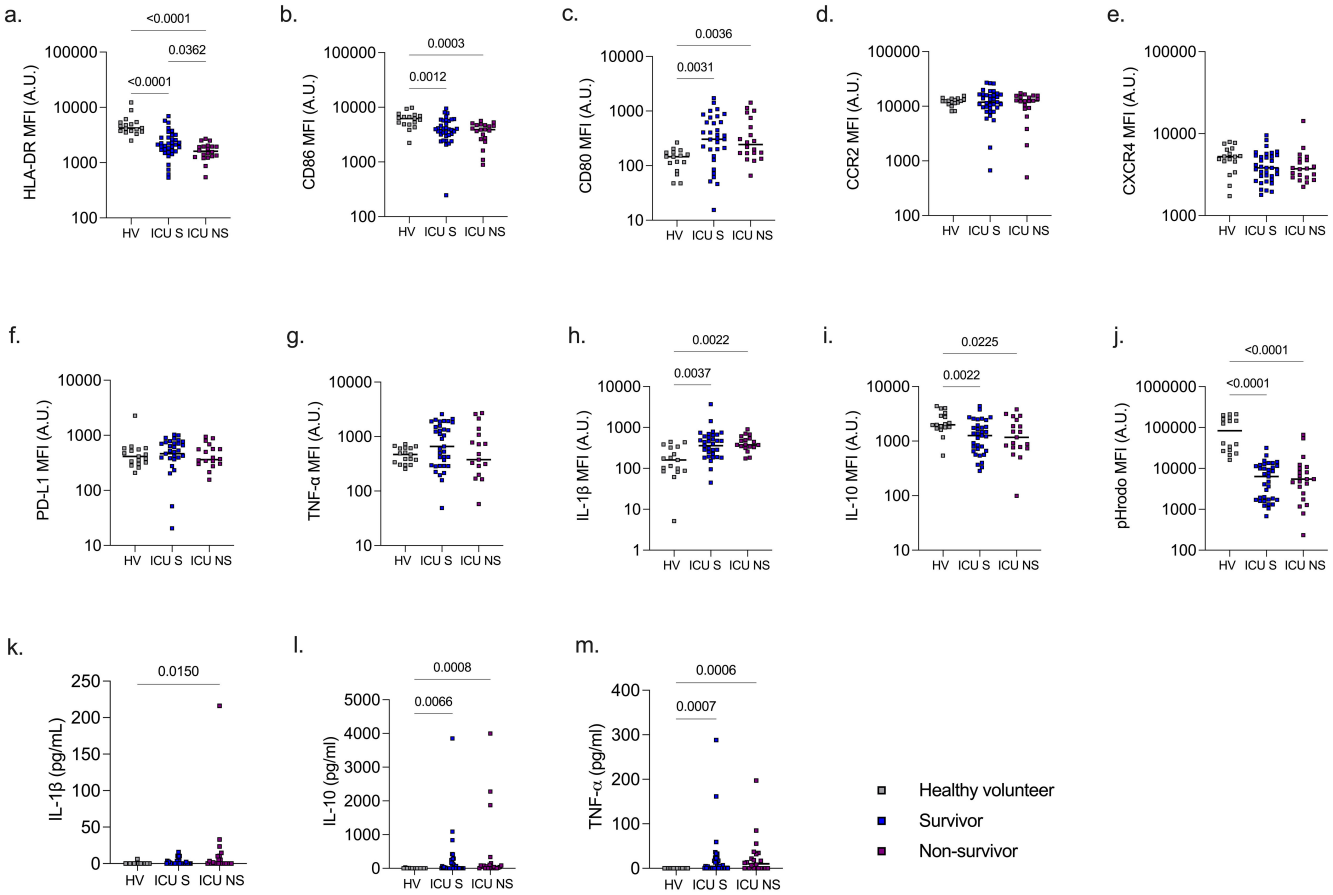
Monocyte phenotype and serum cytokines in healthy volunteers and ICU patient with sepsis. Monocyte HLA-DR **(A)**, CD86 **(B)**, CD80 **(C)**, CCR2 **(D)**, CXCR4 **(E)**, PD-L1 **(F)**, TNF-α **(G)**, IL-1β **(H)**, IL-10 **(I)**, and phagocytosis **(J)** were significantly different among ICU patients compared to healthy volunteers. Only monocyte HLA-DR **(A)** was significantly different between ICU survivors and non-survivors. Serum levels of IL-1β were higher in ICU non-survivors compared to healthy volunteers **(K)** whereas serum IL-10 **(L)** and TNF-α **(M)** were higher both in ICU survivors and on-survivors compared to healthy volunteers.

At baseline, ICU patients exhibited lower levels of monocyte HLA-DR, CD86, IL-10, and phagocytic capacity compared to healthy volunteers, while levels of CD80 and IL-1β were elevated (p <0.05 for all). Among these markers, only monocyte HLA-DR expression distinguished eventual ICU survivors from non-survivors (p = 0.036). Serum cytokine analysis showed elevated IL-10 and TNF-α levels in ICU patients relative to healthy controls, whereas IL-1β was elevated only in non-survivors (p <0.05 for all) (**Figure 1**).

**Figure 2:**
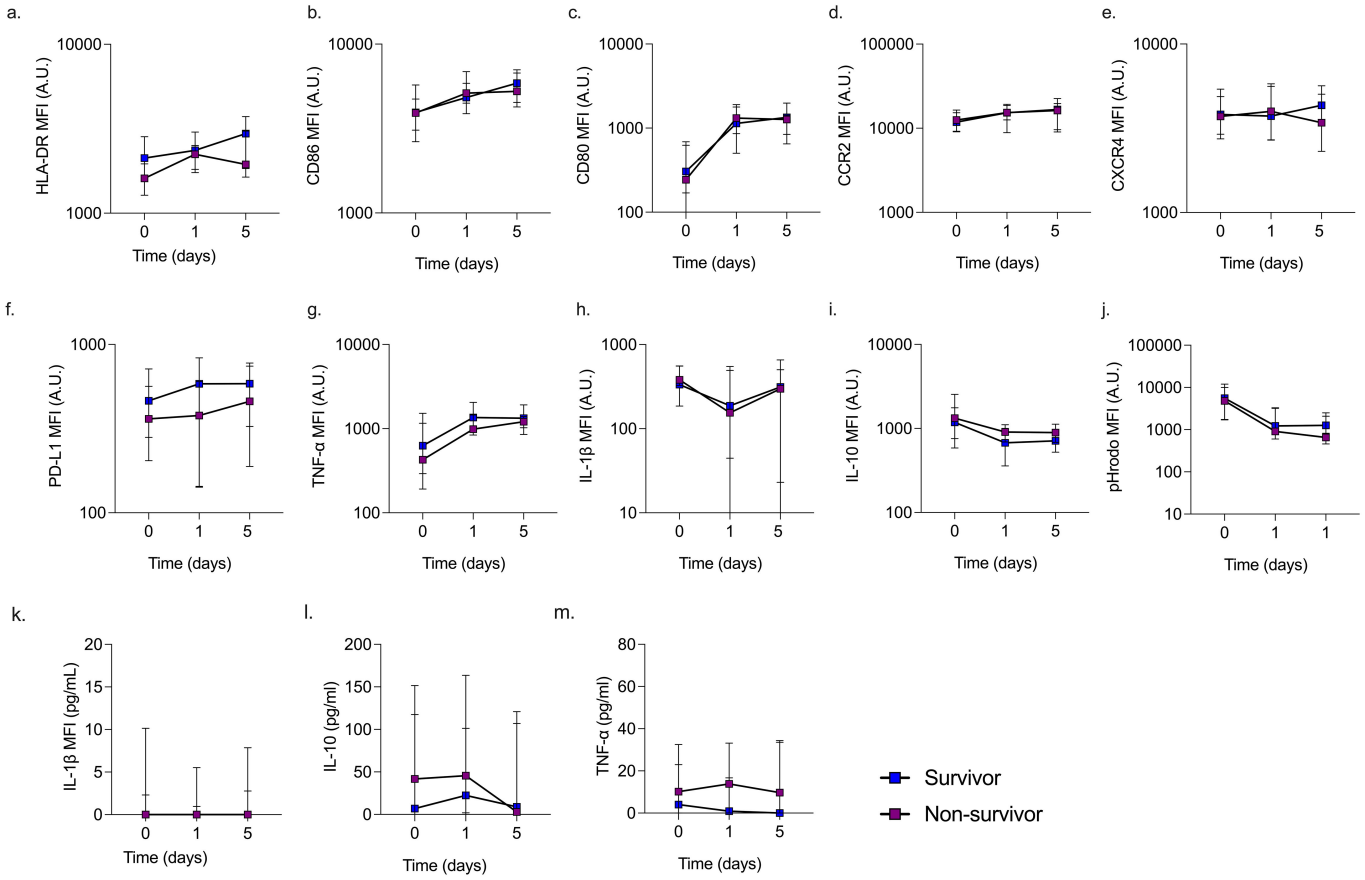
Change in monocyte phenotype and serum cytokines over time in ICU patients. The change in monocyte phenotype **(A–J)** and serum cytokines **(K–M)** over 5 days was similar between ICU survivors and non-survivors, apart from HLA-DR **(A)** which was significantly greater in survivors compared to non-survivors at all time points assessed.

Additional samples were obtained after 1 and 5 days from ICU survivors (n = 24 on day 1 and n = 19 on day 5) and non-survivors (n = 14 on day 1 and n = 8 on day 5). Monocyte HLA-DR (p = 0.0174) and CD86 (p = 0.0011) expression levels increased over time in both survivors and non-survivors, approaching the levels observed in healthy volunteers. In contrast, monocyte IL-10 (p = 0.0008) and phagocytic capacity (p = 0.0041) declined further over time in both groups, whereas CD80 (p <0.0001) continued to rise. Monocyte IL-1β levels remained unchanged throughout the study (**Figure 2**).

Although baseline TNF-α and CCR2 expression were similar between healthy controls and ICU patients, the expression of TNF-α (p <0.0001) and CCR2 (p = 0.0026) increased over time in ICU survivors and non-survivors. The rate of HLA-DR increase was similar between survivors and non-survivors, although survivors exhibited higher HLA-DR expression over 5 days (p = 0.0171).

### Dynamic responses to LPS

**Figure 3:**
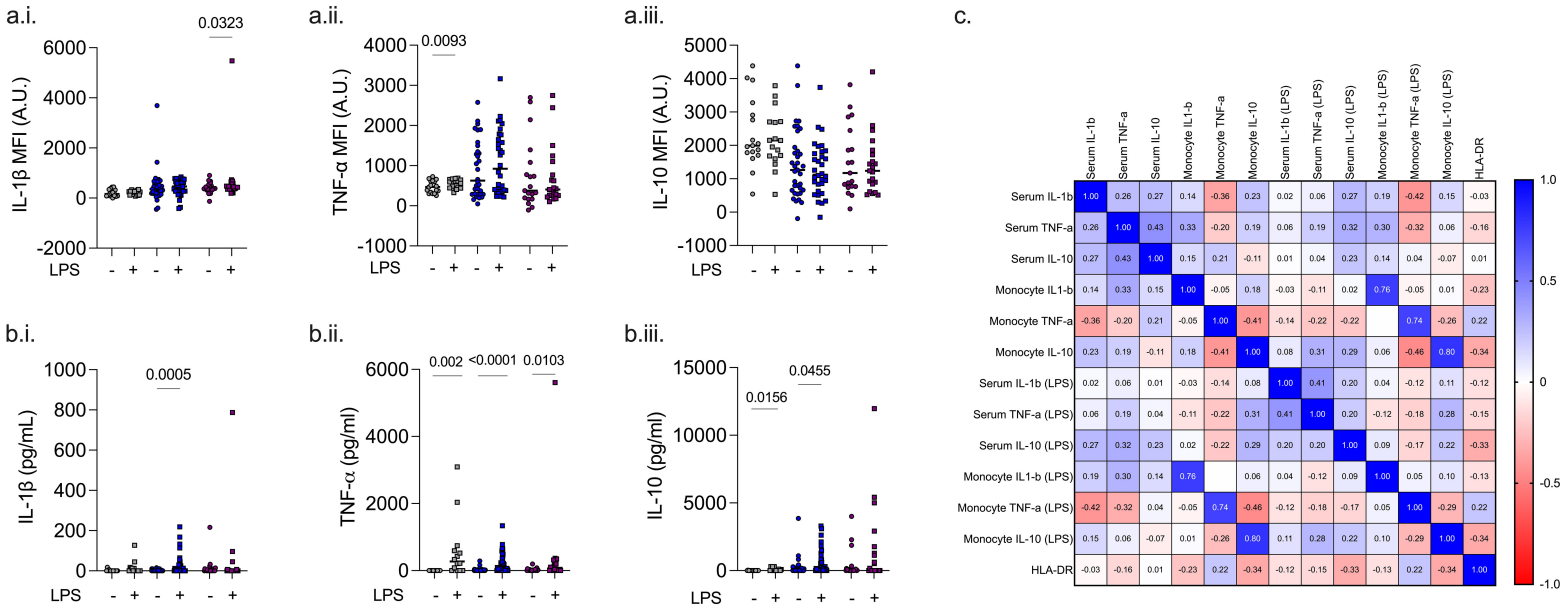
LPS stimulation of PBMCs or whole blood. Intracellular cytokines IL-1β, TNF-α, and IL-10 in healthy volunteer (HV) and patient monocytes following LPS stimulation of PBMCs **(A)**. Serum cytokines IL-1β, TNF-α, and IL-10 in healthy volunteer and patient whole blood following LPS stimulation **(B)**. Correlation between monocyte HLA-DR, released cytokines and monocyte intracellular cytokines before and after LPS stimulation **(C)**.

Following whole blood stimulation with LPS, an increase in serum IL-1β (p = 0.0005) was observed only among ICU survivors, an increase in IL-10 was observed in healthy volunteers (p = 0.016) and ICU survivors (p = 0.046), and an increase in TNF-α was observed in all three cohorts (p <0.01 for all). In contrast, following PBMC incubation with LPS, an increase in monocyte IL-1β (p = 0.032) was observed only in ICU non-survivors, whereas an increase in monocyte TNF-α (p = 0.009) was observed only in healthy volunteers (**Figures 3A, B**).

There was no significant correlation between serum and intracellular cytokine levels, either at baseline or following LPS stimulation. There was a significant correlation between monocyte intracellular Il-1β (r = 0.761; p = 5 × 10^−14^), TNF-α (r = 0.799; p = 4.5 × 10^−13^), and IL-10 (r = 0.742; p = 3.4 × 10^−16^) before and after LPS stimulation (**Figure 3C**). Monocyte HLA-DR levels correlated modestly with monocyte IL-10 (r = −0.339; p = 0.016), LPS-stimulated serum IL-10 (r = −0.328; p = 0.025), and LPS-stimulated monocyte IL-10 (r = −0.337; p = 0.019) levels.

### Internalization of monocyte surface HLA-DR following phagocytosis *ex vivo*

**Figure 4:**
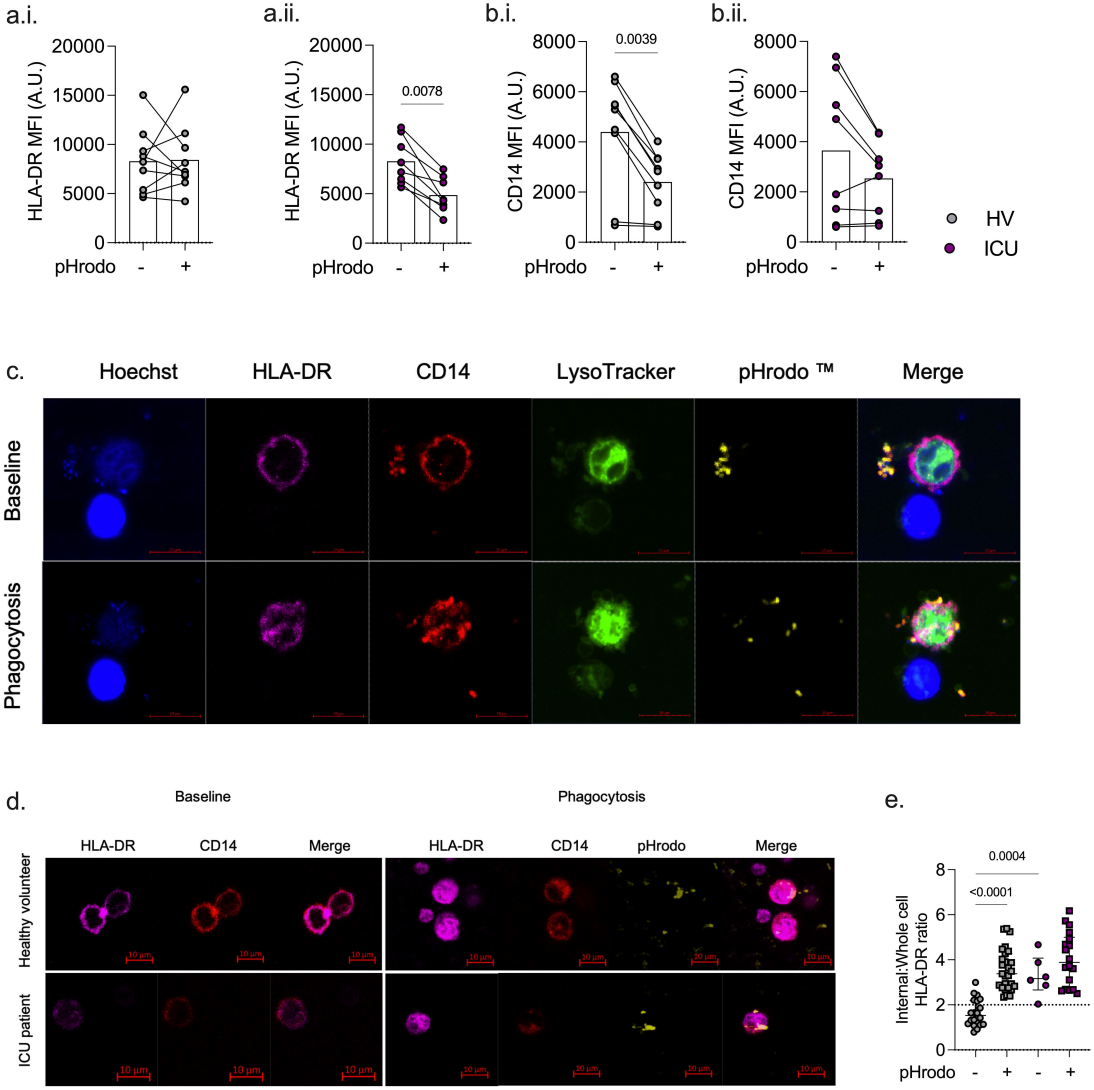
Effect of infection and inflammation on monocyte HLA-DR and phagocytosis. Following phagocytosis of *E coli* bioparticles, monocyte HLA-DR is unchanged in healthy volunteers (HV) but lower in ICU patients as assessed on flow cytometry **(A)**. CD14 levels are lower in healthy volunteers but not in ICU patients following phagocytosis **(B)**. Confocal analysis demonstrates internalization of surface monocyte HLA-DR and CD14 on phagocytosis in healthy volunteer monocytes **(C)**. ICU patient and healthy volunteer monocyte CD14 and HLA-DR localization during phagocytosis assessed using confocal microscopy **(D)**. Healthy volunteer monocyte surface HLA-DR is localized to the cell membrane prior to phagocytosis but is internalized following phagocytosis. ICU patient monocyte HLA-DR is localized within the cell at baseline without significant change on phagocytosis **(E)**.

Immediately following phagocytosis, monocyte surface HLA-DR expression was unchanged in healthy volunteers but was significantly lower in ICU patients (p = 0.008) (**Figure 4A**). Following phagocytosis, surface monocyte CD14 was lower in healthy volunteers (p = 0.004), and there was a trend towards lower CD14 in ICU monocytes, albeit not statistically significant in ICU patient monocytes (**Figure 4B**). Confocal imaging revealed that in unstimulated healthy volunteer monocytes, HLA-DR and CD14 (LPS co-receptor), which are primarily co-localized to the cell membrane, were internalized during phagocytosis (**Figure 4C**). The relative proportion of internalized HLA-DR following phagocytosis increased in healthy volunteer monocytes (p <0.001) but not in ICU patients, where the baseline internalized HLA-DR was higher (compared to healthy volunteers) before phagocytosis (p = 0.0004) (**Figures 4D, E**).

### Reduction in monocyte HLA-DR expression is associated with broad defects in canonical monocyte functional markers

**Figure 5:**
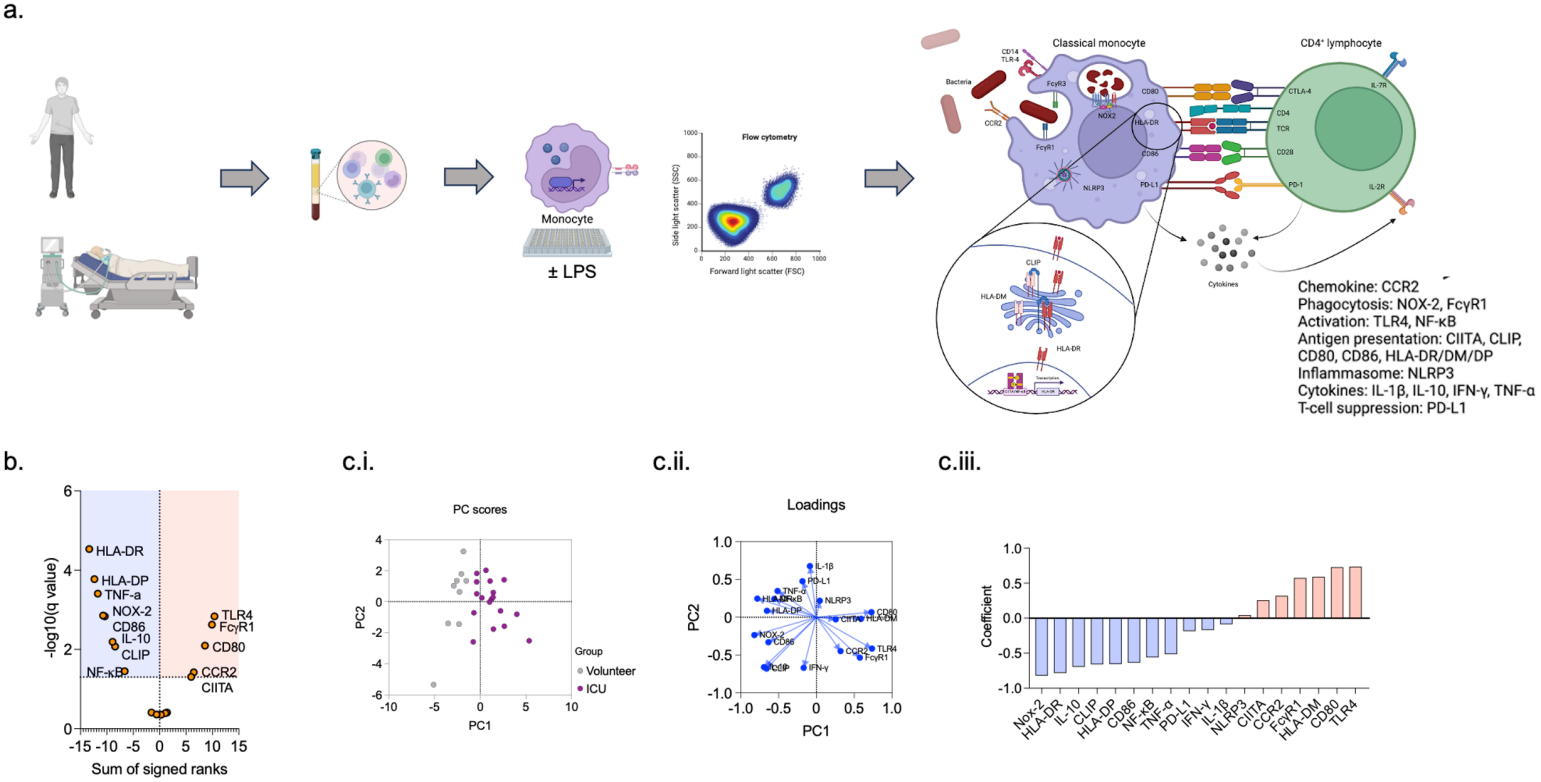
Comparison of healthy volunteer and ICU patient immunophenotype. Compared to healthy volunteer monocytes, monocytes from ICU patients express significantly lower levels of proteins associated with antigen processing and presentation **(A, B)**. Patient and healthy volunteer monocytes are distinguishable on PC analysis, with NOX-2, and HLA-DR providing the greatest variation **(Ci–iii)**.

PBMCs from a subset of healthy volunteers (n = 9) and ICU patients (n = 24) (**Supplementary Table S4**) were used for in-depth immunophenotyping and functional assays (**Figure 5A**). Compared to monocytes from healthy volunteers, monocytes from patients in the ICU with sepsis had a significantly lower expression of proteins associated with the antigen presentation pathway (HLA-DR, HLA-DP, HLA-DR antigen-associated invariant chain (CD74)) and co-stimulation (CD86), cytokines (TNF-α and IL-10), NOX-2, and NF-kB (p65) (adjusted p <0.05 for all). CIITA (MHC II transactivator), CD80, TLR4 (CD284), and Fc receptor (CD64) expression was higher in ICU patient monocytes (adjusted p <0.05 for all) (**Figure 5B**).

Principal component analysis demonstrated separation between healthy volunteers and ICU patients, with several proteins related to antigen presentation (HLA-DR, HLA-DM, HLA-DR, and antigen-associated invariant chain (CD74)) and co-stimulation (CD80, CD86) discriminating between the two groups. Other proteins that demonstrated good discrimination were NOX-2 and TLR4 (**Figure 5C**).

**Figure 6:**
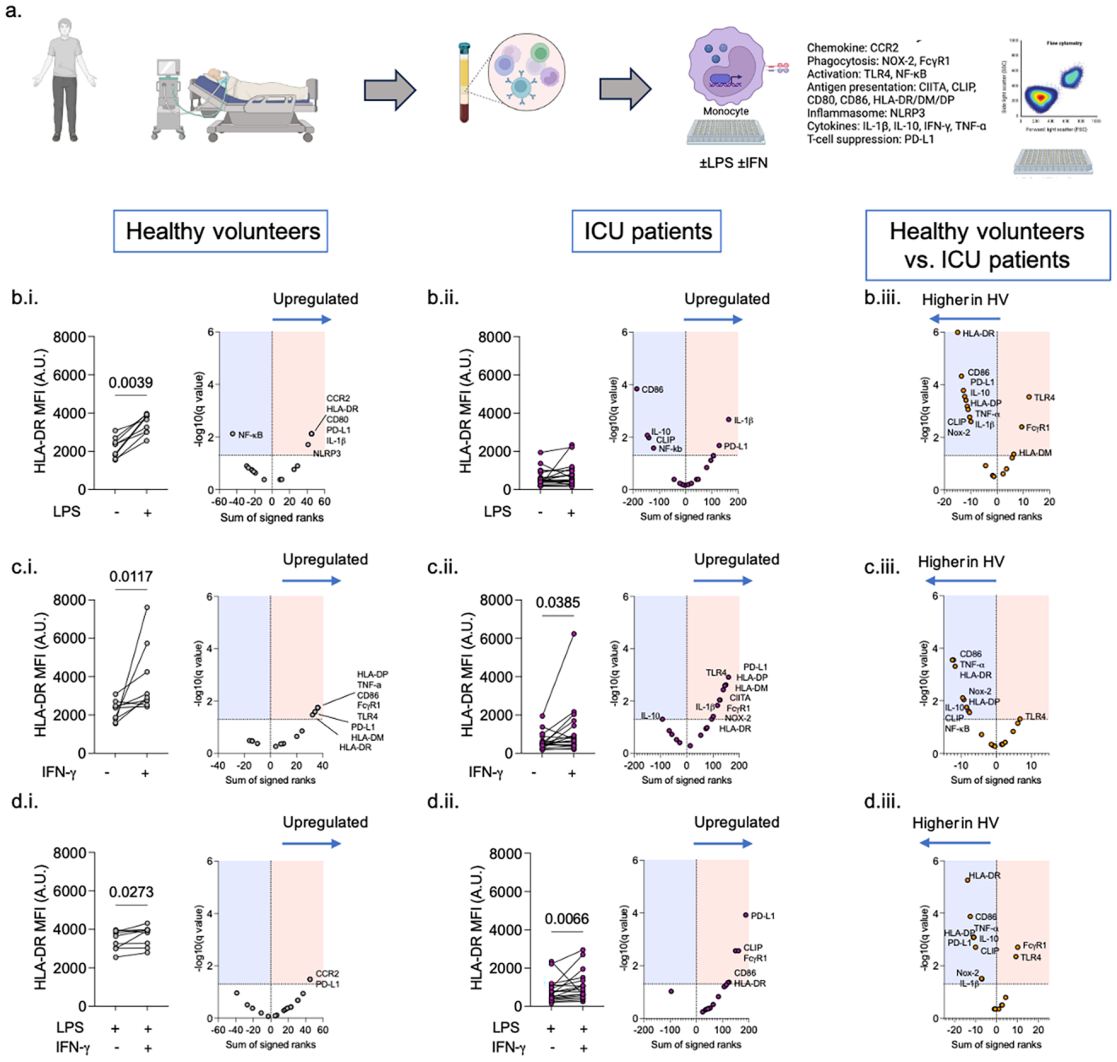
Assessment of monocyte response to LPS and IFN-γ. Experimental plan **(A)**. HLA-DR increases in healthy volunteer monocytes **(Bi)** but not ICU patient monocytes **(Bii)** exposed to LPS. Following LPS exposure, HLA-DR expression remains significantly lower in ICU patient monocytes compared to healthy volunteer monocytes **(Biii)**. HLA-DR increases in healthy volunteer monocytes **(Ci)** and ICU patient monocytes **(Cii)** exposed to IFN-γ. Following IFN-γ. exposure, HLA-DR expression remains significantly lower in ICU patient monocytes compared to healthy volunteer monocytes **(Ciii)**. Combination of LPS and IFN-γ increases monocyte HLA-DR in healthy volunteers on univariate but not multivariate analysis **(Di)**, and increases ICU monocytes patient HLA-DR **(Dii)**. However, HLA-DR remains significantly lower among ICU patient monocytes compared to healthy volunteer monocytes co-stimulated with LPS and IFN-γ **(Diii)**.

Monocytes isolated from ICU patients demonstrated a partial *ex vivo* response to LPS and IFN-γ compared to healthy volunteers (**Figure 6A**).

Following 24 h of LPS exposure, healthy volunteer monocytes demonstrated an increase in HLA-DR, CCR2 (CD192), CD80, (PD-L1 (CD274)), IL-1β, and NLRP3, but reduced NF-κB (p65) expression (adjusted p <0.05 for all) (**Figure 6Bi**). ICU patient monocytes demonstrated an increase in PD-L1 and intracellular IL-1β, with decreased expression of CD86, IL-10, CD74, and NF-κB compared to unstimulated cells (adjusted p <0.05 for all) (**Figure 6Bii**). Compared to LPS-stimulated healthy volunteer monocytes, LPS-stimulated ICU monocytes had higher expression of PD-L1, Fc receptor (CD64), and HLA-DM. However, the expression of several proteins, especially those involved in the antigen presentation and co-stimulation pathways (HLA-DR, HLA-DP, CD86, and CD74), was significantly lower than that in healthy volunteers (adjusted p <0.05 for all) (**Figure 6Biii**).

Following 24 h of exposure to IFN-γ, proteins associated with antigen presentation and co-stimulation pathways were increased in healthy volunteer monocytes (HLA-DR, HLA-DM, HLA-DP, and CD86) (**Figure 6Ci**) and ICU patient monocytes (CIITA, HLA-DM, HLA-DP, and HLA-DR) (**Figure 6Cii**) (adjusted p <0.05 for all). However, compared to healthy volunteer monocytes, the expression of HLA-DR, HLA-DP, CD74, and several other proteins remained significantly lower among ICU patient monocytes (adjusted p <0.05 for all) (**Figure 6Ciii**).

LPS and IFN-γ co-stimulation was associated with an increase in monocyte HLA-DR expression in healthy volunteers (p = 0.027) (**Figure 6Di**) and ICU patients (p = 0.007) (**Figure 6Dii**) (unadjusted analysis). On multivariate analysis, LPS and IFN-γ co-stimulation was associated with an increase in healthy volunteer monocyte CCR2 and PD-L1 (**Figure 6Di**) and ICU patient monocyte PD-L1, CD74, Fc Receptor, CD86, and HLA-DR (**Figure 6Dii**) (adjusted p <0.05 for all). However, the expression of several proteins remained significantly lower in monocytes from ICU patients than in healthy volunteers, including markers associated with antigen presentation and co-stimulation pathways (CD86, HLA-DP, CD74, and HLA-DR), NOX-2, cytokines (IL-1β, IL-10, TNF-α), and CD274 (**Figure 6Diii**) (adjusted p <0.05 for all).

Release of IL-1β was evident in both healthy volunteer (p = 0.008) and patient (p = 0.002) PBMCs stimulated with LPS alone, but not with IFN-γ alone. Release of TNF-α was only evident in healthy volunteers (p = 0.014) PBMCs stimulated with the combination of LPS and IFN-γ. The addition of IFN-γ to LPS-stimulated samples did not further increase TNF-α or IL-1β release in healthy volunteers or ICU patient PBMCs (**Supplementary Figure S4**).

### Temporal dynamics of peripheral blood and tissue monocyte HLA-DR expression

**Figure 7:**
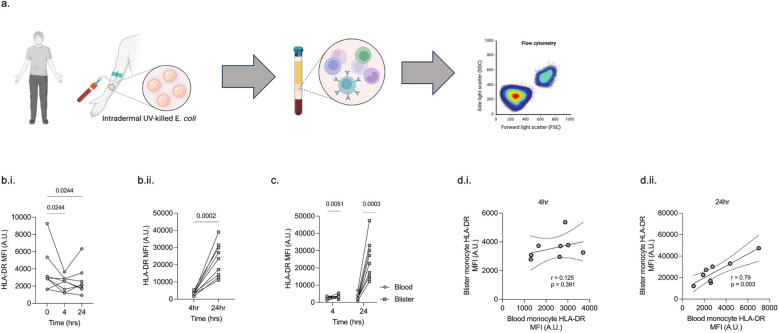
Peripheral blood and blister monocyte HLA-DR expression in healthy subjects administered intradermal LPS. Experimental outline **(A)**. Peripheral blood monocyte HLA-DR is reduced within 4 h of intradermal LPS, which persists at 24 h **(Bii)**. A significant increase in blister fluid monocyte HLA-DR occurs between 4 h and 24 h **(Bii)**. At 4 h and 24 h, the expression of monocyte HLA-DR is significantly higher in blister fluid compared to peripheral blood **(C)**. No correlation exists between peripheral blood monocyte HLA-DR and blister monocyte HLA-DR at 6 h **(Di)**, but at 24 h, correlation is significant **(Dii)**.

Following intradermal administration of UV-killed *E. coli* to healthy volunteers (**Figure 7A**), peripheral blood monocyte HLA-DR was significantly reduced at 4 h (p = 0.024) and 24 h (p = 0.024) compared to baseline levels (**Figure 7Bi**). However, blister fluid monocyte HLA-DR significantly increased from 4 h to 24 h (p = 0.0002) (**Figure 7Bii**) and was significantly higher than PBMC levels at 4 h (p = 0.005) and 24 h (p = 0.0003) (**Figure 7C**). There was a significant positive correlation between peripheral and blister monocyte HLA-DR levels at 24 h (r = 0.79; p = 0.003) but not at 4 h (**Figures 7Di, ii**).

## Discussion

We confirmed previous findings, strengthening the external validity of our data, while also presenting several novel observations. Monocyte phenotypes evolved discordantly over time; some markers trended toward healthy volunteer levels, while others diverged further, with no clear differences between survivors and non-survivors. This highlights the limitations of using a single marker as a prognostic or therapeutic indicator of sepsis.

Dynamic functional testing showed that LPS-induced cytokine release did not predict eventual ICU survival, and serum and intracellular monocyte cytokines were not correlated. Impaired intracellular membrane trafficking following bacterial phagocytosis contributes to reduced surface HLA-DR expression in sepsis. Although IFN-γ administration increased the levels of proteins involved in antigen processing and presentation (including HLA-DR) in ICU patient monocytes, the levels remained significantly lower than those in healthy volunteer monocytes. Finally, in a healthy volunteer *in vivo* model of local inflammation, we demonstrated the temporal evolution of HLA-DR expression in both peripheral blood and tissue monocytes.

Monocyte HLA-DR expression was lower among eventual ICU non-survivors than among survivors. Reduced expression of monocyte HLA-DR is associated with an increase in CD80 but a reduction in CD86 expression (27). CD86 is an important target for immune regulation and control of lymphocyte CD28 co-stimulation, whereas CD80 may attenuate lymphocyte CTLA-4 function through altered trafficking (28). Reduced monocyte HLA-DR and CD86 suggest that the monocyte phenotype in sepsis may be driving an anti-inflammatory phenotype in T-lymphocytes; supporting the utility of monocyte HLA-DR as a biomarker to identify patients with sepsis immunoparalysis. In our study, HLA-DR measurement at ICU admission distinguished between eventual ICU survivors and non-survivors. However, serial measurements are valuable, as differences in monocyte HLA-DR expression may become evident at a later time point (29).

Others have demonstrated that the rate of change of monocyte HLA-DR expression over time may predict eventual survival in ICU patients (30). We found that the expression of HLA-DR over 5 days was significantly higher among survivors than among non-survivors, although the rate of change in monocyte HLA-DR over time was similar between eventual survivors and non-survivors. This difference may be related to several other factors that regulate monocyte HLA-DR in sepsis, including the activity of the Class II transactivator (CIITA) (31) and glucocorticoids (via the suppression of CIITA transcription) (32). Additionally, IL-10 causes a reduction in monocyte surface HLA-DR via endocytosis and intracellular sequestration (33, 34), which explains the inverse correlation between monocyte HLA-DR and IL-10.

The changes in monocyte HLA-DR and other biomarkers over time did not occur in tandem. For example, while monocyte HLA-DR levels increased to levels seen in healthy volunteers, the phagocytic capacity of monocytes decreased in parallel. Others have also found a discordance between the change in biomarkers associated with immunosuppression over time, which underscores the difficulty in using any single marker as a prognostic or therapeutic indicator (35).

While the expression of cell surface receptors is often used as a proxy for immune cell activation or function, the dynamic response of immune cells to stimuli (e.g., LPS) is particularly relevant in the context of secondary infections. The use of *ex vivo* LPS-induced cytokine release in whole blood has been proposed as a surrogate marker of dynamic immune function in sepsis. However, the ability of LPS-induced cytokine production to predict ICU survival remains unclear (36–39). We did not find any association between LPS responses in whole blood cytokine levels, monocyte intracellular cytokines, and eventual survival. Other dynamic measures of immunocompetence may be valuable, including the ELISpot test (40, 41).

Confocal imaging revealed HLA-DR internalization following *E. coli* phagocytosis in healthy volunteer monocytes, with no overall change in monocyte HLA-DR expression as determined by flow cytometry. Taken together, these findings suggest that surface HLA-DR is internalized in monocytes of healthy volunteers following phagocytosis, followed by cell membrane trafficking of *de novo* produced HLA-DR. In contrast, monocytes from ICU patients demonstrated a greater proportion of internalized HLA-DR (relative to membrane-associated HLA-DR) at the baseline. Following *ex vivo* phagocytosis, there was an overall reduction in monocyte surface HLA-DR in ICU patients. In addition, we demonstrated a lower expression of monocyte CD14 in patients with sepsis, as previously described (42, 43).

The components of antigen presentation (HLA-DR, HLA-DP, CD74, and CLIP) and the co-stimulatory pathway (CD86) were downregulated in patients with sepsis in the ICU compared to healthy volunteers. CLIP, generated from cleavage of CD74 (MHC class II invariant chain), is required for the transport of HLA-DR to the cell surface (44). Lower membrane-associated HLA-DR in patients with sepsis, both at baseline and following *ex vivo* phagocytosis, is associated with a lower expression of proteins associated with antigen presentation, such as class II-associated Ii peptide (CLIP) and HLA-DM, despite higher levels of CIITA and FCγ-R1. This suggests that impaired membrane trafficking of intracellular HLA-DR following the internalization of surface HLA-DR during phagocytosis may contribute to reduced monocyte surface HLA-DR expression in sepsis.

NADPH-oxidase (NOX-2) expression was lower, consistent with pHrodo assays demonstrating reduced phagocytosis in ICU patients compared to that in healthy volunteers. However, the finding of reduced phagocytosis is not consistent across reported studies (37, 45), which may be related to the timing of the assessment.

Several proteins that regulate monocyte function are regulated at the transcriptional level; gene expression associated with phagocytosis, antigen presentation, inflammatory response, and cell migration is lower in sepsis than in healthy controls (45). Preliminary data suggest that a multitude of transcriptional changes may be long-lasting following recovery from sepsis (46).

Monocytes isolated from ICU patients had minimal response to *ex vivo* LPS stimulation, which was significantly improved with IFN-γ treatment. IFN-γ treatment aims to restore broader monocyte functionality. HLA-DR serves as an enrichment or stratification marker, but not as a mechanistic endpoint of therapy. Therefore, we assessed several of the proteins required for antigen processing and presentation (including HLA-DR) and other monocyte functional pathways that were increased by exogenous IFN-γ administration. The expression of these proteins was abrogated in the presence of LPS, and their levels were significantly lower than those in monocytes from healthy volunteers. Marginal improvements in functional markers and immune responses with IFN-γ administration were reported in an early clinical trial investigating the role of therapeutic IFN-γ in sepsis (20).

Although IFN-γ treatment increases the expression of several proteins associated with monocyte function in sepsis, it is unclear whether this results in the global restoration of immune function *in vivo*. Reports from published data on monocyte functional responses following *in vivo* IFN-γ treatment are conflicting (20, 21, 47), which likely represents the heterogeneity of patient groups and timing of intervention. In our cohort, *ex vivo* IFN-γ stimulation in monocytes from ICU patients demonstrated heterogeneous effects, with approximately half of the patients responding robustly, while others showed minimal HLA-DR increase. This pattern aligns with the known sepsis heterogeneity and has implications for responder stratification consistent with approaches to identify subtypes or endotypes in patients with sepsis (48, 49). Personalized therapy guided by monocyte HLA-DR surface expression in sepsis-induced immunosuppression shows promise and warrants further evaluation (50).

Additionally, the concurrent increase in PD-L1 with IFN-γ administration may have an untoward immunosuppressive effect on T-cells, although this was not associated with impaired T cell responses *in vitro* in healthy volunteer PBMCs (26).

Circulating classical monocytes migrate to inflamed tissues within 24 h, with the release of classical monocytes from the bone marrow responsible for the restoration of circulating monocytes (51). In sepsis, the expression of HLA-DR from the bone marrow monocyte lineage is reduced in ICU patients compared to non-ICU patients, which occurs in the absence of any significant changes in the phenotype of immature forms of myeloid cells (52). The local inflammatory environment further influences the phenotype of monocytes, which may not resemble circulating cells (compartmentalization of the immune response in sepsis) (53).

Higher alveolar monocyte HLA-DR levels than peripheral blood monocyte HLA-DR levels have been reported in patients with ARDS (36). However, the temporal evolution of concurrent peripheral blood and tissue monocyte HLA-DR expression has not been previously described. Monocyte HLA-DR levels in inflammatory blister fluid were significantly higher than those in peripheral blood at 4 h and 24 h. Although we found s significant positive correlation between peripheral blood and tissue monocyte HLA-DR expression, this occurred only later point. It is unknown how therapeutic interventions based on peripheral blood immune cell phenotypes influence the phenotype and function of cells at the site of inflammation/infection.

Despite the breadth of the data presented, we acknowledge the limitations of this study. Healthy volunteers used as a “reference” for monocyte immunophenotyping were not age-matched with the patient population. However, age-related alterations in HLA-DR expression are relevant at the extremes of age (54, 55). Among our cohort, the correlation between monocyte HLA-DR expression and age was not significant in either healthy volunteers or patients. (**Supplementary Figure S5**) Flow cytometry quantifies the amount of HLA-DR expressed per cell (in this experiment, expressed as MFI). This was independent of the number of monocytes in the sample. The number of monocytes in the bloodstream does not affect the monocyte HLA-DR MFI quantified by flow cytometry. Data on intermediate and non-classical monocyte subsets are not presented as cell counts from patients. Flow cytometry was performed using different analyzers for different experimental protocols. However, we did not make any direct comparisons of MFI values between the experimental protocols. We assessed the levels of several ligands and receptors using flow cytometry but were unable to fully assess their functional relevance or associated pathways.

Cryopreservation of PBMCs may affect the cell surface expression and functional responses of monocytes. However, the expression of monocyte HLA-DR may not change significantly after cryopreservation of PBMCs (56). Additionally, all samples were processed in a similar manner, minimizing any heterogeneity due to processing artifacts. We conducted functional assays in PBMCs instead of isolated monocytes, which may confound the results due to mixed-cell signaling. This is particularly relevant in critical illness, where the PBMC composition may include variable levels of regulatory T-cells or myeloid-derived suppressor cells.

Due to the limited number of cells acquired from patients, we did not assess the effect of G-CSF or GM-CSF, which mechanistically differ from IFN-γ and do not share a unified downstream signaling pathway. The reduction of monocyte HLA-DR expression occurs in critical illness and is not specific to the underlying etiology. Indeed, we have previously demonstrated a significant reduction in monocyte HLA-DR expression within 24 h of major surgery in the absence of infection (24). Although some studies have demonstrated differences in the expression of monocyte HLA-DR depending on the causative organism (57), such results have not been reproduced in all studies (29). We recognize that the *ex vivo* healthy volunteer experiments conducted to evaluate concurrent peripheral blood and tissue monocyte HLA-DR expression were primarily performed as part of a separate study. However, valuable insights from these experiments will help inform future studies related to critically ill patients.

In summary, using an iterative approach of complementary *ex vivo* dynamic functional assays, we provided novel insights into the regulation of monocyte HLA-DR and broader monocyte function in sepsis. Reduced monocyte HLA-DR expression in sepsis reflects broad disruptions across multiple pathways, limiting the efficacy of therapeutic interventions. Further insights into the mechanisms governing therapeutic modulation of monocyte HLA-DR and immune function are required to identify patients who are most likely to benefit from intervention.

## Data Availability

The raw data supporting the conclusions of this article will be made available by the authors, without undue reservation.

## Group members of UCLH critical care research team

Alessia V. Waller, Deborah Smyth, Georgia Bercades, Ingrid Hass, Alexandra Zapata Martinez, Laura Gallagher, and Gladys Martir.

## References

[1] Singer M Deutschman CS Seymour CW Shankar-Hari M Annane D Bauer M . The third international consensus definitions for sepsis and septic shock (Sepsis-3). JAMA. (2016) 315:801–10. doi: 10.1001/jama.2016.0287, PMID: 26903338 PMC4968574

[2] Rudd KE Johnson SC Agesa KM Shackelford KA Tsoi D Kievlan DR . Global, regional, and national sepsis incidence and mortality, 1990-2017: analysis for the Global Burden of Disease Study. Lancet. (2020) 395:200–11. doi: 10.1016/S0140-6736(19)32989-7, PMID: 31954465 PMC6970225

[3] Boomer JS To K Chang KC Takasu O Osborne DF Walton AH . Immunosuppression in patients who die of sepsis and multiple organ failure. JAMA. (2011) 306:2594–605. doi: 10.1001/jama.2011.1829, PMID: 22187279 PMC3361243

[4] Vincent JL Rello J Marshall J Silva E Anzueto A Martin CD . International study of the prevalence and outcomes of infection in intensive care units. Jama. (2009) 302:2323–9. doi: 10.1001/jama.2009.1754, PMID: 19952319

[5] Shankar-Hari M Datta D Wilson J Assi V Stephen J Weir CJ . Early PREdiction of sepsis using leukocyte surface biomarkers: the ExPRES-sepsis cohort study. Intensive Care Med. (2018) 44:1836–48. doi: 10.1007/s00134-018-5389-0, PMID: 30291379

[6] Conway Morris A Anderson N Brittan M Wilkinson TS McAuley DF Antonelli J . Combined dysfunctions of immune cells predict nosocomial infection in critically ill patients. Br J Anaesth. (2013) 111:778–87. doi: 10.1093/bja/aet205, PMID: 23756248

[7] Conway Morris A Datta D Shankar-Hari M Stephen J Weir CJ Rennie J . Cell-surface signatures of immune dysfunction risk-stratify critically ill patients: INFECT study. Intensive Care Med. (2018) 44:627–35. doi: 10.1007/s00134-018-5247-0, PMID: 29915941 PMC6006236

[8] Landelle C Lepape A Voirin N Tognet E Venet F Bohe J . Low monocyte human leukocyte antigen-DR is independently associated with nosocomial infections after septic shock. Intensive Care Med. (2010) 36:1859–66. doi: 10.1007/s00134-010-1962-x, PMID: 20652682

[9] Monneret G Lepape A Voirin N Bohe J Venet F Debard AL . Persisting low monocyte human leukocyte antigen-DR expression predicts mortality in septic shock. Intensive Care Med. (2006) 32:1175–83. doi: 10.1007/s00134-006-0204-8, PMID: 16741700

[10] Cheron A Floccard B Allaouchiche B Guignant C Poitevin F Malcus C . Lack of recovery in monocyte human leukocyte antigen-DR expression is independently associated with the development of sepsis after major trauma. Crit Care. (2010) 14:R208. doi: 10.1186/cc9331, PMID: 21092108 PMC3220028

[11] Livingston DH Appel SH Wellhausen SR Sonnenfeld G Polk HC Jr . Depressed interferon gamma production and monocyte HLA-DR expression after severe injury. Arch Surg. (1988) 123:1309–12. doi: 10.1001/archsurg.1988.01400350023002, PMID: 3140765

[12] Venet F Textoris J Blein S Rol ML Bodinier M Canard B . Immune profiling demonstrates a common immune signature of delayed acquired immunodeficiency in patients with various etiologies of severe injury. Crit Care Med. (2021) 50(4):565–75. doi: 10.1101/2021.03.12.21253466, PMID: 34534131

[13] Meisel C Schefold JC Pschowski R Baumann T Hetzger K Gregor J . Granulocyte-macrophage colony-stimulating factor to reverse sepsis-associated immunosuppression: a double-blind, randomized, placebo-controlled multicenter trial. Am J Respir Crit Care Med. (2009) 180:640–8. doi: 10.1164/rccm.200903-0363OC, PMID: 19590022

[14] Orozco H Arch J Medina-Franco H Pantoja JP González QH Vilatoba M . Molgramostim (GM-CSF) associated with antibiotic treatment in nontraumatic abdominal sepsis: a randomized, double-blind, placebo-controlled clinical trial. Arch Surg. (2006) 141:150–3. doi: 10.1001/archsurg.141.2.150, PMID: 16490891

[15] Prakash V Arora V Jindal A Maiwall R Sarin SK . Combination of GM CSF and carbapenem is superior to carbapenem monotherapy in difficult-to-treat spontaneous bacterial peritonitis: A randomized controlled trial. Liver Int. (2023) 43:1298–306. doi: 10.1111/liv.15534, PMID: 36748109

[16] Presneill JJ Harris T Stewart AG Cade JF Wilson JW . A randomized phase II trial of granulocyte-macrophage colony-stimulating factor therapy in severe sepsis with respiratory dysfunction. Am J Respir Crit Care Med. (2002) 166:138–43. doi: 10.1164/rccm.2009005, PMID: 12119223

[17] Sun J Zhang X Ma L Yang Y Li X . Clinical study of rhGM-CSF for the treatment of pulmonary exogenous acute respiratory distress syndrome by modulating alveolar macrophage subtypes: A randomized controlled trial. Med (Baltimore). (2023) 102:e33770. doi: 10.1097/MD.0000000000033770, PMID: 37171348 PMC10174386

[18] Bo L Wang F Zhu J Li J Deng X . Granulocyte-colony stimulating factor (G-CSF) and granulocyte-macrophage colony stimulating factor (GM-CSF) for sepsis: a meta-analysis. Crit Care. (2011) 15:R58. doi: 10.1186/cc10031, PMID: 21310070 PMC3221991

[19] Vacheron CH Lepape A Venet F Monneret G Gueyffier F Boutitie F . Granulocyte-macrophage colony-stimulating factor (GM-CSF) in patients presenting sepsis-induced immunosuppression: The GRID randomized controlled trial. J Crit Care. (2023) 78:154330. doi: 10.1016/j.jcrc.2023.154330, PMID: 37267804

[20] Döcke WD Randow F Syrbe U Krausch D Asadullah K Reinke P . Monocyte deactivation in septic patients: restoration by IFN-gamma treatment. Nat Med. (1997) 3:678–81. doi: 10.1038/nm0697-678, PMID: 9176497

[21] Livingston DH Loder PA Kramer SM Gibson UE Polk HC Jr . Interferon gamma administration increases monocyte HLA-DR antigen expression but not endogenous interferon production. Arch Surg. (1994) 129:172–8. doi: 10.1001/archsurg.1994.01420260068009, PMID: 7905730

[22] Polk HC Jr Cheadle WG Livingston DH Rodriguez JL Starko KM Izu AE . A randomized prospective clinical trial to determine the efficacy of interferon-gamma in severely injured patients. Am J Surg. (1992) 163:191–6. doi: 10.1016/0002-9610(92)90099-D, PMID: 1739172

[23] Roquilly A Francois B Huet O Launey Y Lasocki S Weiss E . Interferon gamma-1b for the prevention of hospital-acquired pneumonia in critically ill patients: a phase 2, placebo-controlled randomized clinical trial. Intensive Care Med. (2023) 49:530–44. doi: 10.1007/s00134-023-07065-0, PMID: 37072597 PMC10112824

[24] Snow TAC Waller AV Loye R Ryckaert F Cesar A Saleem N . Early dynamic changes to monocytes following major surgery are associated with subsequent infections. Front Immunol. (2024) 15:1352556. doi: 10.3389/fimmu.2024.1352556, PMID: 38655251 PMC11035723

[25] Motwani MP Flint JD De Maeyer RP Fullerton JN Smith AM Marks DJ . Novel translational model of resolving inflammation triggered by UV-killed E. coli. J Pathol Clin Res. (2016) 2:154–65. doi: 10.1002/cjp2.43, PMID: 27499924 PMC4958736

[26] Galbraith NJ Walker SP Gardner SA Bishop C Galandiuk S Polk HC Jr . Interferon-gamma increases monocyte PD-L1 but does not diminish T-cell activation. Cell Immunol. (2020) 357:104197. doi: 10.1016/j.cellimm.2020.104197, PMID: 32891037

[27] Nolan A Kobayashi H Naveed B Kelly A Hoshino Y Hoshino S . Differential role for CD80 and CD86 in the regulation of the innate immune response in murine polymicrobial sepsis. PloS One. (2009) 4:e6600. doi: 10.1371/journal.pone.0006600, PMID: 19672303 PMC2719911

[28] Kennedy A Waters E Rowshanravan B Hinze C Williams C Janman D . Differences in CD80 and CD86 transendocytosis reveal CD86 as a key target for CTLA-4 immune regulation. Nat Immunol. (2022) 23:1365–78. doi: 10.1038/s41590-022-01289-w, PMID: 35999394 PMC9477731

[29] Monneret G Lafon T Gossez M Evrard B Bodinier M Rimmele T . Monocyte HLA-DR expression in septic shock patients: insights from a 20-year real-world cohort of 1023 cases. Intensive Care Med. (2025) 51:1820–32. doi: 10.1007/s00134-025-08110-w, PMID: 40986015 PMC12504386

[30] Wu JF Ma J Chen J Ou-Yang B Chen MY Li LF . Changes of monocyte human leukocyte antigen-DR expression as a reliable predictor of mortality in severe sepsis. Crit Care. (2011) 15:R220. doi: 10.1186/cc10457, PMID: 21933399 PMC3334765

[31] Ting JP Trowsdale J . Genetic control of MHC class II expression. Cell. (2002) 109 Suppl:S21–33. doi: 10.1016/S0092-8674(02)00696-7, PMID: 11983150

[32] Le Tulzo Y Pangault C Amiot L Guilloux V Tribut O Arvieux C . Monocyte human leukocyte antigen-DR transcriptional downregulation by cortisol during septic shock. Am J Respir Crit Care Med. (2004) 169:1144–51. doi: 10.1164/rccm.200309-1329OC, PMID: 15028560

[33] Fumeaux T Pugin J . Role of interleukin-10 in the intracellular sequestration of human leukocyte antigen-DR in monocytes during septic shock. Am J Respir Crit Care Med. (2002) 166:1475–82. doi: 10.1164/rccm.200203-217OC, PMID: 12406851

[34] Mazer M Unsinger J Drewry A Walton A Osborne D Blood T . IL-10 has differential effects on the innate and adaptive immune systems of septic patients. J Immunol. (2019) 203:2088–99. doi: 10.4049/jimmunol.1900637, PMID: 31501258 PMC7206829

[35] Samuelsen A Lehman E Burrows P Bonavia AS . Time-dependent variation in immunoparalysis biomarkers among patients with sepsis and critical illness. Front Immunol. (2024) 15:1498974. doi: 10.3389/fimmu.2024.1498974, PMID: 39712015 PMC11659229

[36] Bendib I Beldi-Ferchiou A Schlemmer F Maitre B Surenaud M Hüe S . Functional ex vivo testing of alveolar monocytes in patients with pneumonia-related ARDS. Cells. (2021) 10(12):3546. doi: 10.3390/cells10123546, PMID: 34944055 PMC8700060

[37] Santos SS Carmo AM Brunialti MK MaChado FR Azevedo LC Assunção M . Modulation of monocytes in septic patients: preserved phagocytic activity, increased ROS and NO generation, and decreased production of inflammatory cytokines. Intensive Care Med Exp. (2016) 4:5. doi: 10.1186/s40635-016-0078-1, PMID: 26879814 PMC4754229

[38] Drewry AM Ablordeppey EA Murray ET Beiter ER Walton AH Hall MW . Comparison of monocyte human leukocyte antigen-DR expression and stimulated tumor necrosis factor alpha production as outcome predictors in severe sepsis: a prospective observational study. Crit Care. (2016) 20:334. doi: 10.1186/s13054-016-1505-0, PMID: 27760554 PMC5072304

[39] Bidar F Bodinier M Venet F Lukaszewicz AC Brengel-Pesce K Conti F . Concomitant assessment of monocyte HLA-DR expression and ex vivo TNF-α Release as markers of adverse outcome after various injuries-insights from the REALISM study. . J Clin Med. (2021) 11(1):96. doi: 10.3390/jcm11010096, PMID: 35011836 PMC8745266

[40] Walton AH Mazer MB Remy KE Osborne DF Davitt EB Griffith TS . Determining potential immunomodulatory drug efficacy in sepsis using ELISpot. Sci Rep. (2025) 15:13464. doi: 10.1038/s41598-025-92016-6, PMID: 40251188 PMC12008245

[41] Price AD Becker ER Barrios EL Mazer MB McGonagill PW Bergmann CB . Surviving septic patients endotyped with a functional assay demonstrate active immune responses. Front Immunol. (2024) 15:1418613. doi: 10.3389/fimmu.2024.1418613, PMID: 39469706 PMC11513262

[42] Venet F Pachot A Debard AL Bohe J Bienvenu J Lepape A . Human CD4+CD25+ regulatory T lymphocytes inhibit lipopolysaccharide-induced monocyte survival through a Fas/Fas ligand-dependent mechanism. J Immunol. (2006) 177:6540–7. doi: 10.4049/jimmunol.177.9.6540, PMID: 17056586

[43] Brunialti MK Martins PS Barbosa de Carvalho H MaChado FR Barbosa LM Salomao R . TLR2, TLR4, CD14, CD11B, and CD11C expressions on monocytes surface and cytokine production in patients with sepsis, severe sepsis, and septic shock. Shock. (2006) 25:351–7. doi: 10.1097/01.shk.0000217815.57727.29, PMID: 16670636

[44] Pinet V Vergelli M Martin R Bakke O Long EO . Antigen presentation mediated by recycling of surface HLA-DR molecules. Nature. (1995) 375:603–6. doi: 10.1038/375603a0, PMID: 7540726

[45] Xu PB Lou JS Ren Y Miao CH Deng XM . Gene expression profiling reveals the defining features of monocytes from septic patients with compensatory anti-inflammatory response syndrome. J Infect. (2012) 65:380–91. doi: 10.1016/j.jinf.2012.08.001, PMID: 22885911

[46] Gritte RB Souza-Siqueira T Borges da Silva E Dos Santos de Oliveira LC Cerqueira Borges R Alves HHO . Evidence for monocyte reprogramming in a long-term postsepsis study. Crit Care Explor. (2022) 4:e0734. doi: 10.1097/CCE.0000000000000734, PMID: 35928539 PMC9345639

[47] Nalos M Santner-Nanan B Parnell G Tang B McLean AS Nanan R . Immune effects of interferon gamma in persistent staphylococcal sepsis. Am J Respir Crit Care Med. (2012) 185:110–2. doi: 10.1164/ajrccm.185.1.110, PMID: 22210794

[48] Tang G Luo Y Song H Liu W Huang Y Wang X . The immune landscape of sepsis and using immune clusters for identifying sepsis endotypes. Front Immunol. (2024) 15:1287415. doi: 10.3389/fimmu.2024.1287415, PMID: 38707899 PMC11066285

[49] Mekata K Kyo M Tan M Shime N Hirohashi N . Molecular endotypes in sepsis: integration of multicohort transcriptomics based on RNA sequencing. J Intensive Care. (2025) 13:30. doi: 10.1186/s40560-025-00802-1, PMID: 40448231 PMC12123803

[50] Leventogiannis K Kyriazopoulou E Antonakos N Kotsaki A Tsangaris I Markopoulou D . Toward personalized immunotherapy in sepsis: The PROVIDE randomized clinical trial. Cell Rep Med. (2022) 3:100817. doi: 10.1016/j.xcrm.2022.100817, PMID: 36384100 PMC9729870

[51] Patel AA Zhang Y Fullerton JN Boelen L Rongvaux A Maini AA . The fate and lifespan of human monocyte subsets in steady state and systemic inflammation. J Exp Med. (2017) 214:1913–23. doi: 10.1084/jem.20170355, PMID: 28606987 PMC5502436

[52] Faivre V Lukaszewicz AC Payen D . Downregulation of blood monocyte HLA-DR in ICU patients is also present in bone marrow cells. PloS One. (2016) 11:e0164489. doi: 10.1371/journal.pone.0164489, PMID: 27893741 PMC5125574

[53] Conway Morris A Rynne J Shankar-Hari M . Compartmentalisation of immune responses in critical illness: does it matter? Intensive Care Med. (2022) 48:1617–20. doi: 10.1007/s00134-022-06871-2, PMID: 36050558 PMC9436168

[54] Wu J Liu Z Zhang Y Wang L Feng D Liu L . Age-dependent alterations of HLA-DR expression and effect of lipopolysaccharide on cytokine secretion of peripheral blood mononuclear cells in the elderly population. Scand J Immunol. (2011) 74:603–8. doi: 10.1111/j.1365-3083.2011.02612.x, PMID: 21854407

[55] Busse S Steiner J Alter J Dobrowolny H Mawrin C Bogerts B . Expression of HLA-DR, CD80, and CD86 in healthy aging and alzheimer’s disease. J Alzheimers Dis. (2015) 47:177–84. doi: 10.3233/JAD-150217, PMID: 26402766

[56] Appleby LJ Nausch N Midzi N Mduluza T Allen JE Mutapi F . Sources of heterogeneity in human monocyte subsets. Immunol Lett. (2013) 152:32–41. doi: 10.1016/j.imlet.2013.03.004, PMID: 23557598 PMC3684771

[57] Cajander S Rasmussen G Tina E Magnuson A Soderquist B Kallman J . Dynamics of monocytic HLA-DR expression differs between bacterial etiologies during the course of bloodstream infection. PloS One. (2018) 13:e0192883. doi: 10.1371/journal.pone.0192883, PMID: 29466395 PMC5821339

