# Supplementary material for: Challenges of monocyte HLA-DR targeted immunomodulation in sepsis—a prospective observational cohort study
Source: Front Immunol. 2026 Jan 7;16:1709289. doi: 10.3389/fimmu.2025.1709289 (PMC12819683; doi:10.3389/fimmu.2025.1709289)
Supplement: Supplementary file 1 [file DataSheet1.docx]

# Supplemental Tables

| Cell Marker | Catalogue no. | Fluorochrome | Concentration |
| --- | --- | --- | --- |
| CD14 | BL 301840 | BV785 | 1:250 |
| CD16 | BD 563785 | BUV395 | 1:250 |
| HLA-DR | BL 307618 | APC-Cy7 | 1:250 |
| CD80 | BL 305208 | PE | 1:250 |
| CD86 | BL 374218 | PE-Dazzle | 1:250 |
| CD184 (CXCR4) | BL 306518 | BV421 | 1:250 |
| CD192 (CCR2) | BL 357232 | BV711 | 1:250 |
| CD274 (PD-L1) | BD 563741 | APC | 1:250 |
| pHRodo E. *coli* | TF P35361 | PE | 100mcg/mL |
| L/D Blue | TF L34962 | Blue UV | 1:1000 |
| IL-1β | TF 11701842 | FITC | 1:100 |
| IL-6 | BD 561441 | APC | 1:100 |
| IL-10 | BL 501422 | BV421 | 1:100 |
| TNF-α | BL 502930 | PE- Cy7 | 1:100 |

Supplementary Table S1: Flow cytometry fluorochromes. Abbreviations: CCR2: C-C motif chemokine receptor 2; CXCR4: C-X-C motif chemokine receptor 4; CD: cluster of differentiation; IL: Interleukin, PD-L1: Programmed death-ligand 1; BL: Biolegend; BD: Beckton Dickinson; TF: Thermo Fisher.

| Function | Marker type | Marker | Fluorochrome | Catalogue no | Dilution |
| --- | --- | --- | --- | --- | --- |
| Gating | Cell surface | CD14 | SBB580 | MCA1568SBB580 | 1:250 |
| Gating | Cell surface | CD16 | SB702 | 67-0168-42 | 1:250 |
| Gating /Antigen presentation | Cell surface | HLA-DR | BUV805 | 748338 | 1:250 |
| Phagocytosis | Cell surface | CD64 | BUV737 | 612776 | 1:250 |
| Antigen presentation | Cell surface | CD74 | BV650 | 743734 | 1:250 |
| Antigen presentation | Cell surface | CD80 | BV480 | 751735 | 1:250 |
| Antigen presentation | Cell surface | CD86 | BUV496 | 749895 | 1:250 |
| Chemokine receptor | Cell surface | CD192 | BUV395 | 747854 | 1:250 |
| T-cell suppression | Cell surface | CD274 | RB545 | 756359 | 1:250 |
| Toll-like receptor | Cell surface | CD284 | SB600 | 63-9917-42 | 1:250 |
| Antigen presentation | Cell surface | HLA-DM | APC | 130-124-252 | 1:250 |
| Antigen presentation | Cell surface | HLA-DP | RB780 | 755757 | 1:250 |
| Viability | Viability | Live/Dead | Zombie NIR | 423106 | 1:1000 |
| Cytokine | Intracellular cytokine | IL-1b | AF750 | FAB10349S-100UG | 1:100 |
| Cytokine | Intracellular cytokine | IL-10 | BB700 | 566567 | 1:100 |
| Cytokine | Intracellular cytokine | IFN-g | BV750 | 566357 | 1:100 |
| Cytokine | Intracellular cytokine | TNF-a | BV785 | 502948 | 1:100 |
| Antigen presentation | Transcription factor | CIITA | DY680 | NBP2-59072FR | 1:100 |
| Activation | Intracellular protein | NF-kb p65 | PE-CF594 | 565447 | 1:100 |
| Inflammasome | Intracellular protein | NLRP3 | AF405 | IC7578V-100UG | 1:100 |
| Phagocytosis | Intracellular protein | NOX-2 | PE-Cy7 | NBP1-41012PECY7 | 1:100 |

Supplementary Table S2: Spectral Flow cytometry fluorochromes. Abbreviations: CCR2: C-C motif chemokine receptor 2; CIITA: Class II major histocompatibility complex transactivator; CXCR4: C-X-C motif chemokine receptor 4; CD: cluster of differentiation; IL: Interleukin, NIR: Near-infra red; NLRP3: NLR family pyrin domain containing 3; NOX-2: NADPH oxidase 2; PD-L1: Programmed death-ligand 1; BL: Biolegend; BD: Beckton Dickinson; TF: Thermo Fisher.

| Cell Marker | Cat no | Fluorochrome |
| --- | --- | --- |
| CD14 | BL 301830 | BV421 |
| CD16 | BL 302018 | APC-Cy7 |
| HLA-DR | MB 130-111-789 | PE |

**Supplementary Table S3: *In vivo* blister model flow cytometry fluorochromes.**

|  | | Volunteers  (n=9) | ICU  (n=24) |
| --- | --- | --- | --- |
| Age (years) | | 22 (21-41) | 63 (49-74) |
| Sex (male) n (%) | | 5 (56%) | 17 (71%) |
| Ethnicity | | | |
|  | Asian | 5 (56%) | 2 (8%) |
|  | Black | 0 | 1 (4%) |
|  | Middle eastern | 1 (11%) | 0 |
|  | Other/Not Stated | 0 | 6 (25%) |
|  | White | 3 (33%) | 14 (58%) |
| Co-morbidities | | | |
|  | Diabetes | - | 5 (21%) |
|  | COPD | - | 4 (17%) |
|  | Heart failure | - | 3 (13%) |
|  | Ischaemic heart disease | - | 2 (8%) |
| Chronic Immunosuppression | | | |
|  | Biological | - | 1 (4%) |
|  | Steroid | - | 1 (4%) |
| Source of infection | | |  |
|  | Pulmonary | - | 15 (63%) |
|  | GI | - | 6 (25%) |
|  | GU | - | 2 (8%) |
|  | ENT | - | 0 |
|  | Soft tissue | - | 0 |
|  | Bacteraemia | - | 3 (13%) |
|  | Other | - | 1 (4%) |
| Clinical parameters | | | |
|  | Temperature | - | 37.8 (37.1-38.7) |
|  | GCS | - | 15 (15-15) |
|  | Respiratory rate | - | 26 (20-34) |
|  | qSOFA score | - | 1 (1-2) |
| Laboratory values | | | |
|  | WBC (x10^6^) | - | 9.8 (4.1-12.5) |
|  | Neutrophils (x10^6^) | - | 7.6 (3.6-10.5) |
|  | Lymphocytes (x10^6^) | - | 0.90 (0.48-1.46) |
|  | Monocytes (x10^6^) | - | 0.67 (0.46-0.92) |
|  | CRP (mg/l) | - | 148 (53-259) |
|  | Lactate (mmol/l) | - | 0.9 (0.7-1.4) |

**Supplementary Table S4:** Demographics, clinical and laboratory characteristics of healthy volunteers and patients for *ex vivo* LPS ± IFN-y stimulation.

# Supplemental Figures


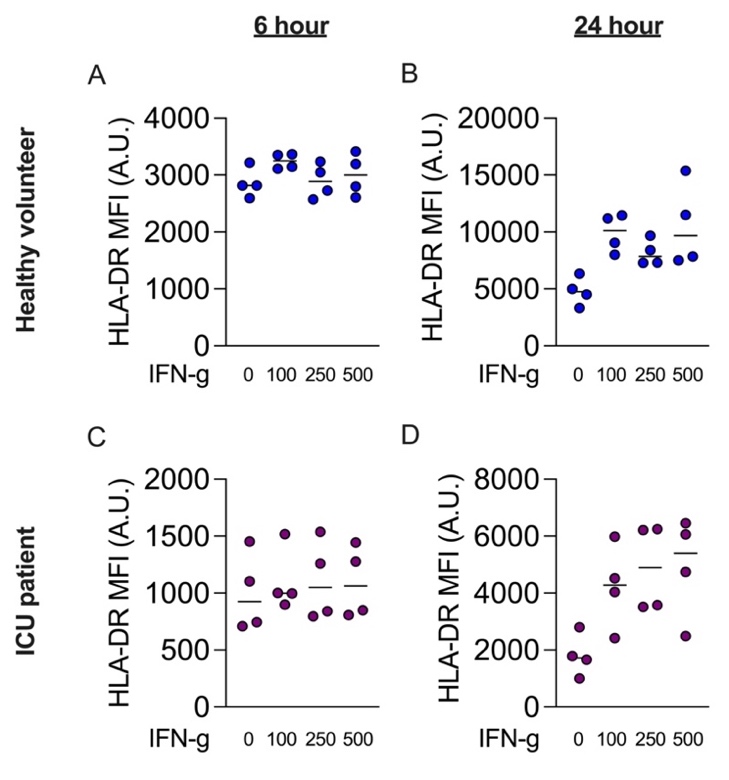


Supplementary Figure S1: Dose titration of IFN-y in healthy volunteer PBMCs. Volunteer PBMCs (n=4) were stimulated three doses of IFN-y (100, 250 and 500ng/ml). (A) Six-hour IFN-y stimulation did not result in an increase in monocyte HLA-DR, irrespective of dose. (B) Following 24- hours of IFN-y exposure, an increase in monocyte HLA-DR was evident with 100ng/mL, with no added effect of higher doses.


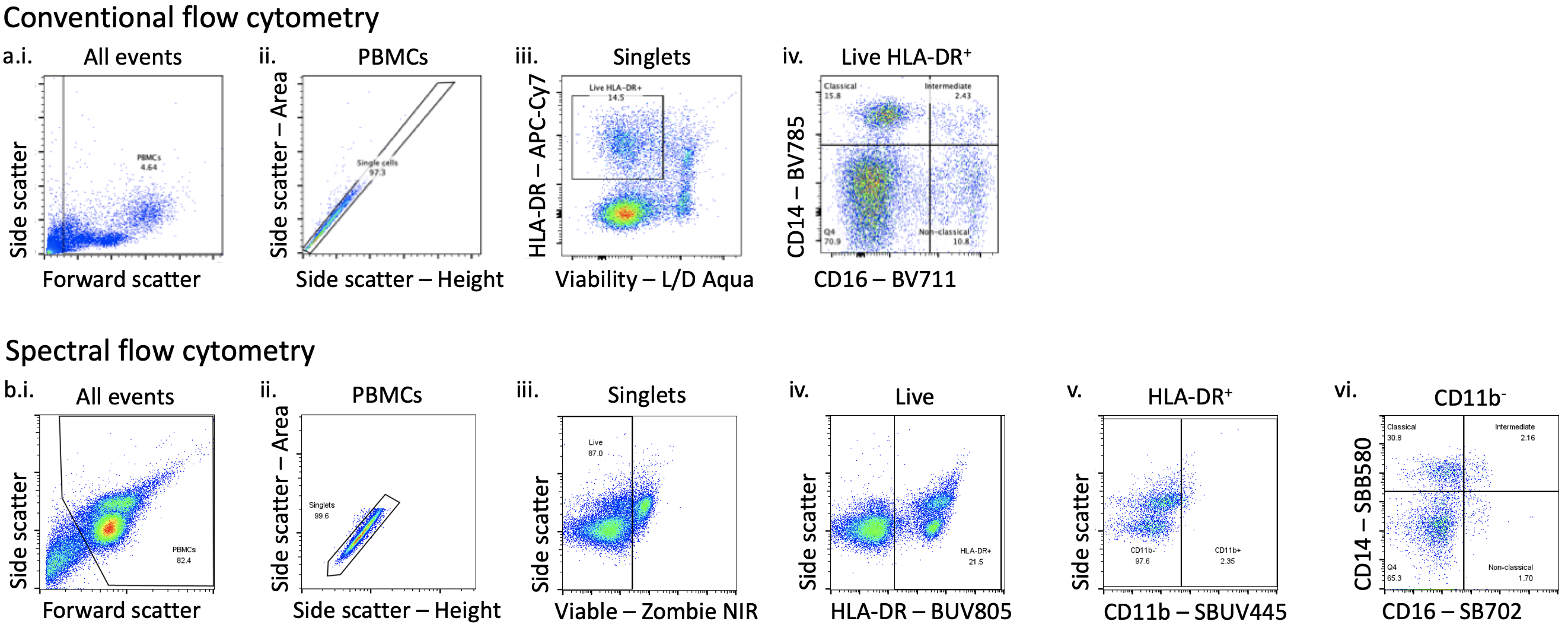


**Supplementary Figure S2**: Gating strategy for monocytes. (a.) Conventional flow cytometry gating by (i.) PBMCs, (ii.) Singlets, (iii.) Live HLA-DR^+^ cells, (iv.) subset differentiation (CD14 and CD16). (b.) Spectral flow cytometry gating by (i.) PBMCs, (ii.) Singlets, (iii.) Live, (iv.) HLA-DR^+^, (v.) CD11b^-^, subset differentiation (CD14 and CD16).

Screened

n = 106

Exclusions:

No staff available n = 1

Declined consent n = 1

Enrolled

n = 104

Exclusions:

Haem-oncology n = 19

Insufficient sample n = 12

No lab staff available n = 8

Immunomodulatory therapy n = 4

Chemotherapy n = 3

Withdrew consent n = 2

Previously recruited n= 1

Included in cohort analysis

n = 55

ICU non-survivor

n = 20

ICU Survivor

n = 35

**Supplemental Figure S3: Consort diagram of recruited patients**


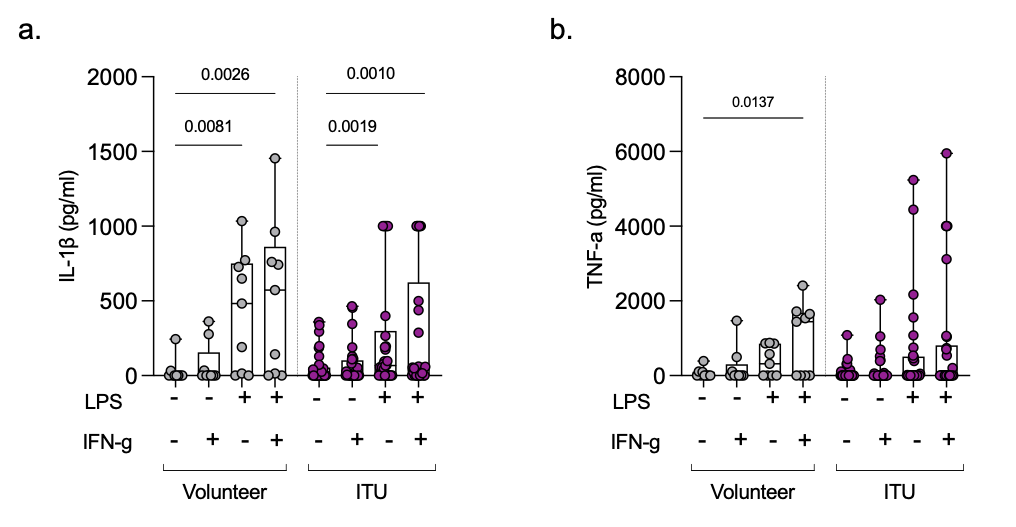


**Supplementary Figure S4:** Cytokine release following 24-hour of LPS (100ng/mL) ± IFN-y (100ng/mL) exposure in PBMC samples obtained from healthy volunteers and ICU patients.

**Supplementary Figure S5:** Correlation between HLA-DR expression and age calculated using simple linear regression for basic immunophenotyping cohort volunteers (i.), ICU patients (ii.), and combined volunteers and patients (iii.).
